# Vessel-based CTA-image to spatial anatomy registration using tracked catheter position data: preclinical evaluation of *in vivo* accuracy

**DOI:** 10.1186/s41747-024-00499-1

**Published:** 2024-08-28

**Authors:** Geir Arne Tangen, Petter Aadahl, Toril A. N. Hernes, Frode Manstad-Hulaas

**Affiliations:** 1 SINTEF Digital, Department of Health Research, Trondheim, Norway; 2 Norwegian National Center for Minimally Invasive and Image-Guided Diagnostics and Therapy, St. Olavs Hospital, Trondheim, Norway; 3 Department of Circulation and Medical Imaging, Norwegian University of Science and Technology, Trondheim, Norway; 4 Department of Cardiothoracic Anesthesia and Intensive Care, St. Olavs Hospital, Trondheim, Norway; 5 Future Operating Room, St. Olav University Hospital, Trondheim, Norway; 6 Department of Radiology, St. Olavs Hospital, Trondheim, Norway

**Keywords:** Aortic aneurysm (abdominal), Computed tomography angiography, Endovascular procedures, Radiation exposure, Surgical navigation systems

## Abstract

**Abstract:**

Electromagnetic tracking of endovascular instruments has the potential to substantially decrease radiation exposure of patients and personnel. In this study, we evaluated the *in vivo* accuracy of a vessel-based method to register preoperative computed tomography angiography (CTA) images to physical coordinates using an electromagnetically tracked guidewire. Centerlines of the aortoiliac arteries were extracted from preoperative CTA acquired from five swine. Intravascular positions were obtained from an electromagnetically tracked guidewire. An iterative-closest-point algorithm registered the position data to the preoperative image centerlines. To evaluate the registration accuracy, a guidewire was placed inside the superior mesenteric, left and right renal arteries under fluoroscopic guidance. Position data was acquired with electromagnetic tracking as the guidewire was pulled into the aorta. The resulting measured positions were compared to the corresponding ostia manually identified in the CTA images after applying the registration. The three-dimensional (3D) Euclidean distances were calculated between each corresponding ostial point, and the root mean square (RMS) was calculated for each registration. The median 3D RMS for all registrations was 4.82 mm, with an interquartile range of 3.53–6.14 mm. A vessel-based registration of CTA images to vascular anatomy is possible with acceptable accuracy and encourages further clinical testing.

**Relevance statement:**

This study shows that the centerline algorithm can be used to register preoperative CTA images to vascular anatomy, with the potential to further reduce ionizing radiation exposure during vascular procedures.

**Key Points:**

- Preoperative images can be used to guide the procedure without ionizing intraoperative imaging.
- Preoperative imaging can be the only imaging modality used for guidance of vascular procedures.
- No need to use external fiducial markers to register/match images and spatial anatomy.
- Acceptable accuracy can be achieved for navigation in a preclinical setting.

**Graphical Abstract:**

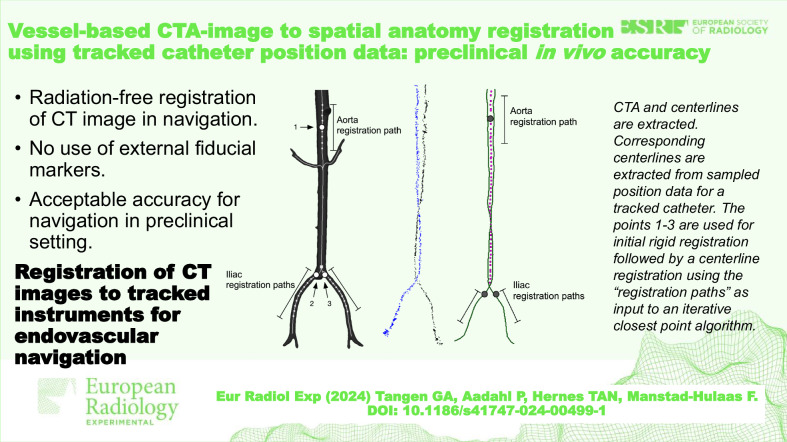

## Background

Endovascular interventions require extensive use of images, both for preoperative planning and guidance during the procedure. This is especially important in complex cases involving fenestrated and branched stent grafts [1]. Intraoperative imaging has mainly been based on two-dimensional fluoroscopy and digital subtraction angiography to visualize and guide instruments and stent graft introducers in real time through vascular anatomy.

In the past decade, the technological development of flat panel detectors has enabled hybrid angiography operating rooms (ORs) with intraoperative cone-beam computed tomography with three-dimensional (3D) visualization [2]. Preoperative images can provide a static, high-resolution 3D map of the complete aorta with branching vessels and serve as guidance for the operator during the navigation of catheters and guidewires, possibly reducing the need for radiation and nephrotoxic contrast agents [3].

The intraoperative use of preoperative 3D images requires sufficiently accurate spatial conformity between the image data and patient anatomy. The process enabling this conformity is referred to as patient registration and has traditionally been performed by using known landmarks in a rigid point-to-point registration [4] or image fusion based on mutual information [5]. Image fusion techniques, matching preoperative high-resolution 3D images to intraoperative imaging, are now state-of-the-art in angiography procedures [6, 7]. Registration is typically performed during the first phase of an interventional procedure. It is relatively time-consuming and influenced by several factors that can potentially reduce accuracy, such as patient movements, respiration, and anatomical shifts during the procedure [8, 9].

To reduce the need for ionizing intraoperative imaging, electromagnetic positioning systems can be used for real-time tracking of catheters and guidewires combined with navigation technology [10]. Catheters and guidewires move inside the vascular anatomy of the blood vessels and the recorded position data will form a point cloud representing the vascular anatomy of the patient. By applying an iterative-closest-point registration method, the resulting point cloud can be matched to the centerline of the corresponding blood vessels identified in the preoperative images. Promising results have previously been reported in phantom studies by Nypan et al [11], with a median registration accuracy of 3.75 mm.

One assumption is that the catheter or guidewire will move along the periphery of the vessel and that the recorded 3D position data can be registered to the closest positions on the vessel wall. This has been done with good results in a phantom study by de Lambert et al [12], with a mean registration error of 1.3 mm. However, our assumption is that this registration technique may be insufficient in an *in vivo* situation where the bloodstream and pulsatile vessel movement may result in accentuated movement of the instruments in the cross-sectional plane of the vessel. Instead, a centerline can be created from the recorded position data point cloud, and an equivalent centerline of the blood vessel in the preoperative 3D image is easily extracted. Based on these centerlines, a registration matching images and spatial anatomy can be performed. A similar centerline registration method has also been suggested in bronchoscopy [13].

The aim of this investigation was to clarify whether the centerline registration provides acceptable accuracy for endovascular navigation procedures in an operating room setup.

## Methods

Data from five domestic swine with a weight of approximately 50 kg was used. The Institutional Animal Care and Use Committee approved the study, and all animals were treated according to the Guide for the Care and Use of Laboratory Animals. The animals were anesthetized in the animal laboratory according to a standardized protocol. A tracheal tube was inserted for ventilation, and a central venous access for the injection of imaging contrast was established. The animal was transported to a computed tomography (CT) scanner, and contrast-enhanced CT angiography (CTA) was acquired before the animal was transferred to a hybrid OR. The animal was placed in the same supine orientation on the OR table as in the CT scanner. A commercial 0.035 in guidewire (Starter Guidewire, Boston Scientific, Quincy, USA) was modified by replacing its steel core with an electromagnetic position sensor and cabling (Aurora 5DOF Sensor, Part No. 610099; Northern Digital Inc., Waterloo, Ontario, Canada). The tip of the guidewire was cut off, and the sensor was placed in the remaining tip, fixated and sealed with epoxy. The guidewire was inserted in the lumen of a 5 French pig-tail catheter, and the combined tool was inserted through a bilateral femoral access. The catheter and guidewire were advanced to the level of the diaphragm and slowly pulled back through the aorta into the iliac artery while tracking and logging the position of the guidewire (sampling rate 30 Hz). The electromagnetic tracking volume was provided by the Aurora Planar 20–20 Field.

The generator was mounted underneath the operating table. Both left and right femoral access introducers were used six times each, providing six sampled position data point clouds per iliac.

### Preoperative image data processing

A dual-energy CT scan (SOMATOM Definition Flash, Siemens Healthineers, Forchheim, Germany) was used, with the following technical parameters: 100 and 140 kVp; 512 × 512 image matrix; resolution 0.59 mm/pixel; slice thickness 1.5 mm, with 1.0 mm slice distance. Intravenous contrast injection (Omnipaque 350 mg I/mL, 30 mL with injection rate 3 mL/s flushed with 30 mL of saline) was performed with the animal in the supine position. A bone-suppression algorithm included in the Siemens software (Syngo.via VA44A, Siemens Healthineers, Forchheim, Germany) was then executed using this dual-energy scan as input. This generated an image series with bone structures removed. This dataset was exported to a digital versatile disc (DVD) for further image processing.

From this image sequence, the aorta, including the left and right renal arteries, superior mesenteric artery, and left and right iliac artery were segmented using the software application ITK-SNAP [14]. Further processing was performed with the Vascular Modeling Toolkit [15]. The output from this step was: (1) a 3D surface mesh of the vascular anatomy and (2) an extracted centerline model of the vascular anatomy.

**Fig. 1:**
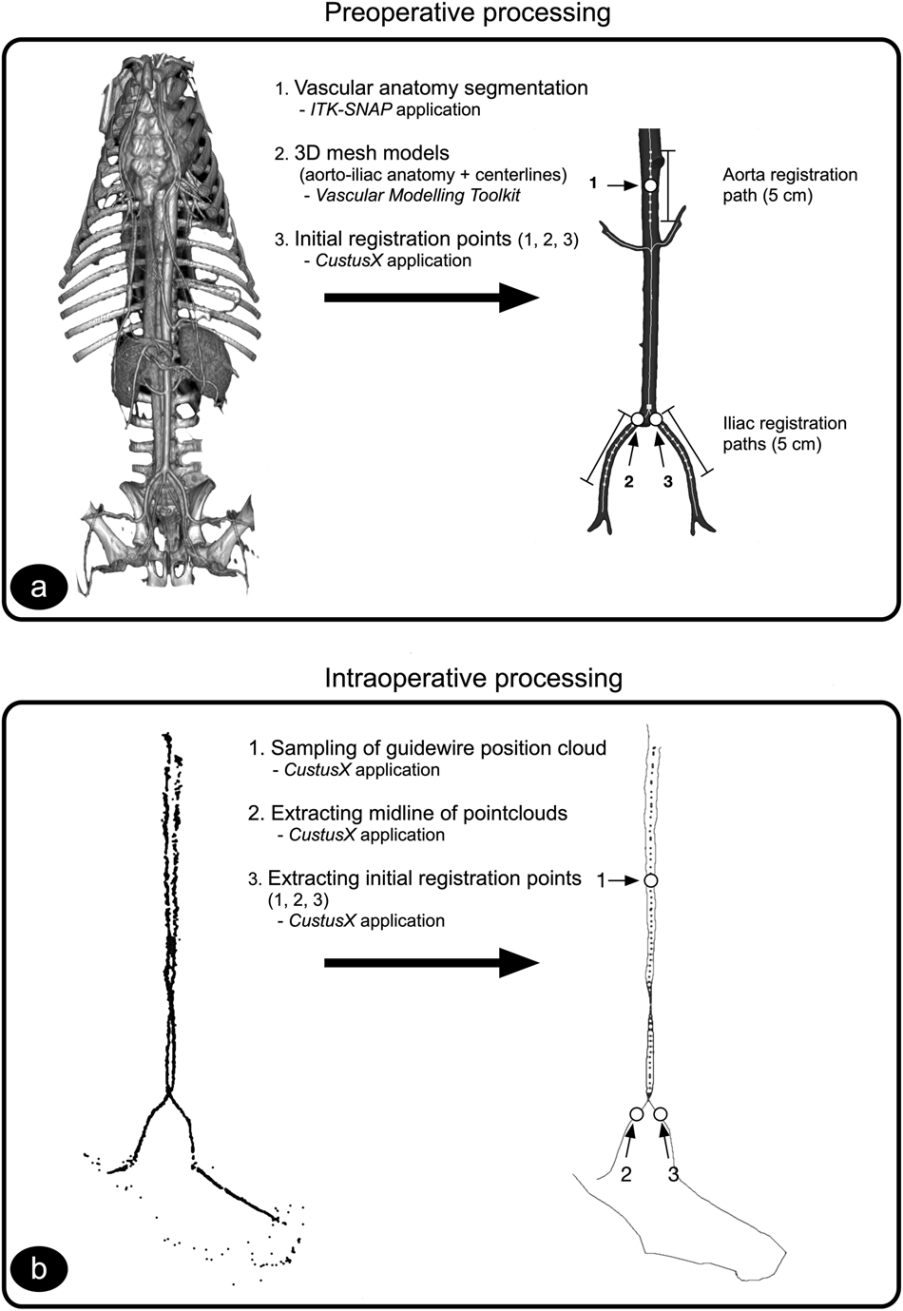
**a** Preoperative processing of data for registration. Extraction of aortic vascular anatomy as three-dimensional mesh models with centerlines, using the software packages ITK SNAP and Vascular Modelling Toolkit. The initial registration points (1, 2, 3), together with the registration paths (Aorta registration path and Iliac registration paths), are extracted. **b** Intraoperative processing of data for registration. Acquisition of position data from an electromagnetically tracked instrument (guidewire/catheter) pulled down the aorta-iliac vascular anatomy on both sides. Centerlines of these point clouds are extracted together with initial registration points (1, 2, 3)

The results from these processing steps were then imported into the CustusX navigation system [16] together with the original CTA Digital Imaging and Communications in Medicine (DICOM) image dataset. The total time used for the preoperative processing of the image data, performed by an experienced medical engineer, was approximately 20 min per animal (Fig. 1a). In CustusX, the interface toward the Aurora position tracking system was handled. Further processing of the sampled guidewire data and the registration process were performed by the CustusX system, as described in the next paragraphs.

### Navigation system processing

During the procedure, the guidewire positions in the abdominal aorta and both iliac arteries were logged by the navigation system and saved as a point cloud (Fig. 1b). As indicated in Fig. 1, the logged position data were preprocessed before being input into the registration algorithm. The centerlines for both the left and right iliac point clouds were extracted, and the two centerlines in the aortic segment were joined by extracting the centroid of the two lines. This process is illustrated by the dotted line in Fig. 1b. Three points, one supra-renal (1), the right (2), and the left (3) iliac ostia, were extracted from the CT centerlines (Fig. 1a). The equivalent three points were then extracted from the position data centerlines (Fig. 1b) and later used for initial rigid registration. The sampling of the guidewire position and further processing added about 30 s to the procedure.

### Vessel-based registration

An algorithm for registration of the centerline of the logged guidewire position data to match the CT centerlines was implemented as a plugin module in the CustusX navigation system. This plugin will be released in the open-source domain as part of the CustusX platform, along with this publication. An iterative-closest-point algorithm [17] using the ITK Registration framework (Insight Segmentation and Registration ToolKit) [18] was used to match the two different pointsets (CT centerline and logged position data). Despite placing the animal in the same supine position in the OR as during CT imaging, some degree of deformation between the two datasets is inevitable. We approached this challenge by using only part of the centerlines (the supra-renal aortic registration path, right and left iliac registration paths, each with a length of 50 mm) in the registration, as illustrated in Fig. 1a. The paths of the upper iliac arteries will presumably have minimal deformation caused by the inserted introducers.

The algorithm was implemented as a two-step process. First, we performed an initial rigid landmark registration based on the three points described above, in the supra-renal aorta (1), the right (2), and the left (3) iliac ostia (see Fig. 1a, b). Then, the registration was refined by performing an iterative-closest-point algorithm using the supra-renal aortic registration path and the right and left iliac registration paths, as indicated in Fig. 1a. The execution of the registration algorithm (initial and refine step) was completed in about 4–5 s.

### Validation

**Fig. 2:**
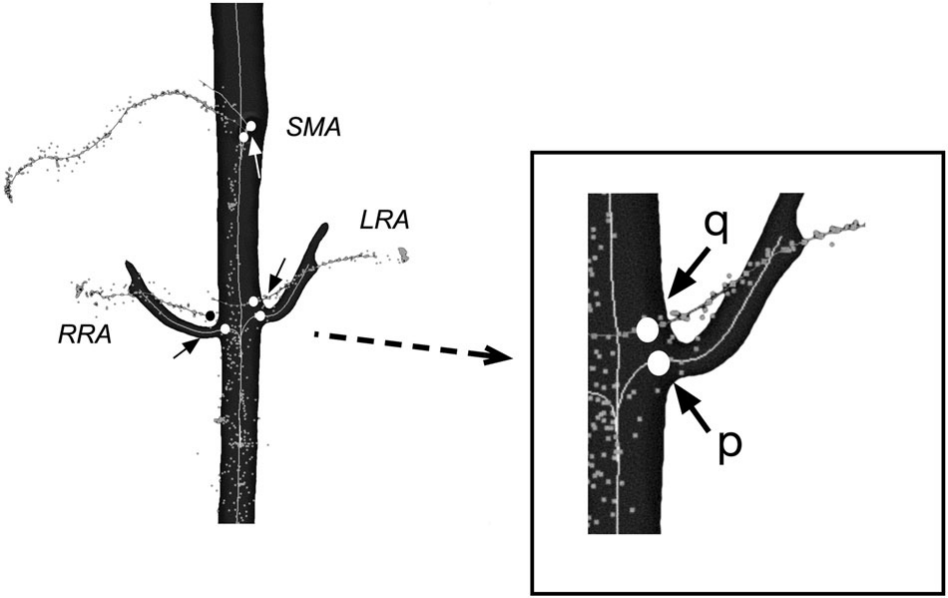
Accuracy calculation. Point clouds for guidewire path tracking with extracted centerlines are shown in light gray. The three-dimensional Euclidean distance between the manually identified ostia (*p*) of the side branches and the nearest point (*q*) on the point-cloud midlines is calculated

For evaluation, we cannulated the side branches of the abdominal aorta (bilateral renal arteries and superior mesenteric artery) with the guidewire and catheter, aided by x-ray fluoroscopy. After placing the catheter and guidewire in one of these branching vessels, the catheter and guidewire were slowly pulled back down into the iliac artery while continuously logging the position data. This process was repeated 1–4 times for each of the side branches, and the registration accuracy was evaluated at the ostia for each of the three side branches (right renal artery, left renal artery, and superior mesenteric artery) as shown in Fig. 2. We calculated the 3D Euclidean distance (1) between each of these points (manually identified in the CTA, point *p*) and the nearest point on the corresponding centerline of the position data point cloud, point *q*.

1 $$d(p,q)=\sqrt{({q}_{1}-{p}_{1})+({q}_{2}-{p}_{2})+({q}_{3}-{p}_{3})}$$

A root mean square (RMS_3D_) (2) was calculated as an accuracy metric for the registration.

2 $${RM}{S}_{3D} = \sqrt{\frac{{\sum }_{n=1}^{3}{(d(p,q))}^{2}}{3}}$$

## Results

**Fig. 3:**
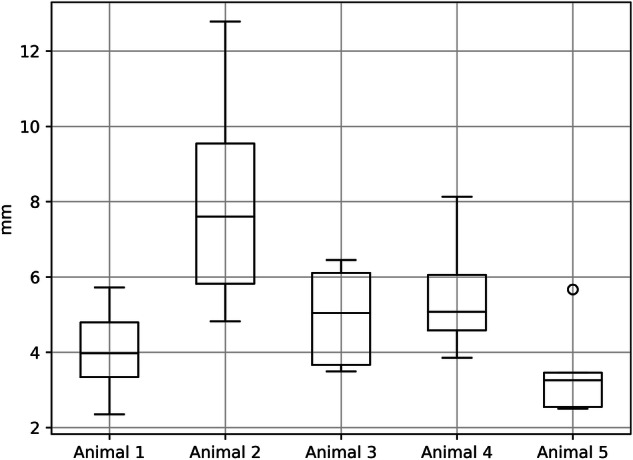
Registration accuracy (root mean square) results for each of the five animals. Results specified in mm. Boxplot is generated by the Python software package pandas v.1.5.2 (pandas.pydata.org)

Successful registration was performed in all cases for the five animals, except for the last registration (registration 6) for animal 5. In this case, the algorithm failed to generate the midline for the corresponding position data point cloud. The position data point cloud from the right renal artery acquisition for animal 1 had several dropouts and could not be used as the basis for validation. For this animal, the RMS accuracy numbers for the registration were based on accuracy calculated in two side-branch ostia (left renal artery and superior mesenteric artery). For the remaining animals, we calculated the accuracy in all three side-branch ostia. The median 3D RMS registration accuracy for all registrations was 4.8 mm. Table 1 and Fig. 3 show detailed accuracy results for the side-branch evaluation points in all five animals.

**Table 1:** Root mean square (RMS) accuracy results (mm)

Registrations	Animal 1	Animal 2	Animal 3	Animal 4	Animal 5	Summary
1	3.6	5.3	6.5	4.7	3.3	
2	4.4	12.8		3.9	5.7	
3	5.7	7.4	3.5	4.6	3.5	
4	3.3	4.8	6.1	6.2	2.6	
5	2.4	10.1	3.5	5.5	2.5	
6	4.9	7.8	6.0	8.1		
Median RMS	4.0	7.6	5.0	5.1	3.3	4.8
Min RMS	2.4	4.8	3.5	3.9	2.5	2.4
Max RMS	5.7	12.8	6.5	8.1	5.7	12.8
Q1	3.3	5.8	3.7	4.6	2.6	3.5
Q3	4.8	9.5	6.1	6.1	3.5	6.1

Registration accuracy (Root mean square) results for each of the five animals. Six registrations (1–6), based on the six tracked pull-back of the guidewire on each side, were performed per animal. Results and descriptive statistics are specified in mm

## Discussion

The registration method performed reasonably well in an operating room setup with anatomical deformation (respiration, blood flow, and introducers in the iliac arteries). Based on the anatomical dimensions of the human aortic side branches, we have considered a positional accuracy of < 5 mm as clinically acceptable [19]. A recent paper by Ivashchenko et al [20], implementing a similar setup using electromagnetic tracking but for a different clinical procedure, obtained a comparable measured navigation accuracy of 4.0 ± 3.0 mm.

Although the animals were placed in the same supine position on the OR table as in the CT scanner, an anatomical deformation may occur that negatively affects the registration between the two centerlines. Our algorithm addresses this challenge by performing a segmental rigid registration in the volume of interest. An alternative approach is using deformable registration [21, 22], which results in a more computationally demanding method that requires realistic and accurate modeling of the deformation. For one of the animals (animal 2), the registration results were more inaccurate and had a larger variation. Investigation of the position point-cloud data showed a quite large sideways deformation of the aorta. This deformation could have been caused by a changed position of the animal on the OR table compared to the CTA. It can be an indication that large anatomical deformation causes unstable performance of the algorithm, even when limiting the registration to a smaller volume of interest.

Patient registration based on real-time position tracking of instruments has the potential to reduce the need for intraoperative fluoroscopy and cone-beam CT or the need for a dedicated preoperative CT with external fiducial markers for landmark registration. This can greatly improve the logistics during the procedure, reduce the time used for registration, and make existing preoperative images available during endovascular guidance. Such a navigation system also has the potential to provide continuous, real-time position data, which enables patient registration to be continuously updated and improved. Since a dedicated image acquisition for navigation purposes is not needed, this can reduce the total accumulated radiation and contrast dose delivered to the patient.

Electromagnetic tracking is susceptible to disturbances by nearby metal and electromagnetic noise. In this study, we addressed this limitation by carefully making sure that metal equipment like the C-arm was moved away from the tracking systems volume of interest. This makes it difficult to use x-ray imaging and electromagnetic tracking simultaneously. Fiber optic shape sensing based on Fiber Bragg Grating Sensors is a tracking technology that is not disturbed by fluoroscopy [23]. This interesting technology can be used in combination with both x-ray and electromagnetic sensors, although the fabrication process is complex and fiber optic cables are sensitive to temperature and strain.

Future improvements to the method may include real-time, continuous updating of the registration. This method can also be extended by integrating shape sensing technology (Fiber Bragg Grating Sensors, etc.) to provide a continuously updated point cloud with position data to the algorithm. Our results indicate that the accuracy of this method is clinically acceptable, though further studies are needed to verify the stability of the registration method in realistic settings. We are planning a study evaluating the performance and accuracy of the algorithm with clinical data from patients with abdominal aortic aneurysms.

To make electromagnetic tracking clinically available, close collaboration with the instrument vendors is needed. Electromagnetic sensors need to be integrated, and the modified instruments must be approved for clinical use (*i.e.*, CE-certified in Europe). An example of a company based in the USA that has such collaboration and sells an electromagnetic-based navigation system for vascular procedures is Centerline Biomedical, (Cleveland, Ohio, centerlinebiomedical.com).

In conclusion, this preliminary study on a swine model showed that a vessel-based registration of CTA images to vascular anatomy is possible with acceptable accuracy and encourages clinical testing on patients.

## Data Availability

The software used for registration in this study is implemented in the open-source platform CustusX and can be downloaded from https://www.custusx.org. Imaging data and raw data from the sampling of position data are available from the corresponding author upon reasonable request.

## Abbreviations

3D: Three-dimensional
CT: Computed tomography
CTA: Computed tomography angiography
OR: Operating room
RMS: Root mean square
